# Anti-tumor effect of local injectable hydrogel-loaded endostatin alone and in combination with radiotherapy for lung cancer

**DOI:** 10.1080/10717544.2020.1869864

**Published:** 2021-01-11

**Authors:** Na Wang, Qin Gao, Juan Tang, YiQing Jiang, LiShi Yang, XiangXiang Shi, Yue Chen, Yan Zhang, ShaoZhi Fu, Sheng Lin

**Affiliations:** a Department of Oncology, Affiliated Hospital of Southwest Medical University, Luzhou, China; b Department of Oncology, Zigong First People’s Hospital, Zigong, China; c Nuclear Medicine and Molecular Imaging Key Laboratory of Sichuan Province, Affiliated Hospital of Southwest Medical University, Luzhou, China

**Keywords:** Endostatin, hyaluronic acid, hydrogel, radiotherapy, lung cancer

## Abstract

Endostatin (ES) can effectively inhibit neovascularization in most solid tumors and has the potential to make oxygen delivery more efficient and increase the efficacy of radiotherapy (RT). With a short half-life, ES is mainly administered systemically, which leads to low intake in tumor tissue and often toxic systemic side effects. In this study, we used hyaluronic acid-tyramine as a carrier to synthesize an ES-loaded hydrogel drug (ES/HA-Tyr) that can be injected locally. ES/HA-Tyr has a longer half-life and fewer systemic toxic side effects, and it exerts a better anti-angiogenic effect and anti-tumor effect with RT. *In vitro*, ES/HA-Tyr showed sustained release in the release assay and a stronger ability to inhibit the proliferation of human umbilical vascular endothelial cells (HUVECs) in the MTT assay; it exhibited a more potent effect against HUVEC invasion and a stronger anti-angiogenic effect on HUVECs in tube formation. *In vivo*, ES/HA-Tyr increased local drug concentration, decreased blood drug concentration, and caused less systemic toxicity. Further, ES/HA-Tyr effectively reduced tumor microvessel density, increased tumor pericyte coverage, decreased tumor hypoxia, and increased RT response. ES/HA-Tyr + RT also had increased anti-tumor and anti-angiogenic effects in Lewis lung cancer (LLC) xenograft models. In conclusion, ES/HA-Tyr showed sustained release, lower systemic toxicity, and better anti-tumor effects than ES. In addition, ES/HA-Tyr + RT enhanced anti-angiogenic effects, reduced tumor hypoxia, and increased the efficacy of RT in LLC-bearing mice.

## 1. Introduction

The morbidity and mortality rates of lung cancer are higher in China than in the rest of the world (Liu et al., 2018; Cao & Chen, 2019). Most patients are diagnosed in the intermediate-advanced stages, with concurrent radiotherapy (RT) and chemotherapy recommended as the main treatment, which significantly limits treatment options.

Hypoxia is a common phenomenon in solid tumors that often hampers the effect of RT (Nordsmark et al., 2005). Unlike normal blood vessels, tumor blood vessels are structurally and functionally defective, which affects oxygen transport (Jain, 2001). Following Folkman’s hypothesis of vascular normalization of tumors, anti-angiogenic therapy has become a key area of research in the past two decades. Recombinant human endostatin (ES) acts by regulating various receptors on the cell membrane and thus inhibits angiogenesis, as well as tumor cell migration and invasion. ES is an endogenous anti-angiogenic peptide with many anti-tumor effects (Qiu et al., 2013; Xiao et al., 2015); it could make oxygen delivery more efficient, alleviate tumor hypoxia, and enhance sensitivity to RT (Jiang et al., 2012; Peng et al., 2012; Meng et al., 2013). However, the main mode of administration of ES is intravenous delivery, and increasing the dose of ES may lead to systemic toxic side effects. Therefore, it is very important to modify ES administration to achieve a higher concentration of this drug in tumor tissues to enhance its anti-tumor effects while also reducing side effects. CT-guided biopsy of lung cancer, a conventional diagnosis technology, provides an opportunity for intratumoral injection using a coaxial puncture needle for drug delivery after the biopsy.

To reduce the systemic side effects and improve its utilization rate by the organism, new drug delivery systems, including nanoparticles, microspheres, targeted drugs, hydrogels, and chemically modified drugs, have been developed (Lee et al., 2009; Li et al., 2009; Zheng et al., 2012; Shi et al., 2014; Li et al., 2016; Hu et al., 2017; Yan et al., 2017; Yun et al., 2017). However, most drug-loaded systems cannot be used clinically due to issues with stability, compatibility, and biosafety. Hyaluronic acid (HA) is a linear mucopolysaccharide consisting of multiple glucuronic acids and N-acetylglucosamine disaccharide units. It is a biodegradable, biocompatible, nontoxic, immunogenic, and non-inflammatory linear polysaccharide, as well as a major constituent of the native extracellular matrix during early human embryogenesis (Toole, 2001; 2004; Xu et al., 2012). Among the natural and synthetic hydrogels, HA hydrogels have attracted much interest, and their chemical, physical, and biological properties have been studied. HA hydrogels have been widely used in osteoarthritis treatment, surgical wound healing, medical beauty, embryo implantation, drug delivery, tissue engineering, etc. (Gao et al., 2010; Lee et al., 2010; Huang et al., 2017; Raia et al., 2017; Sun et al., 2018; Nguyen et al., 2019).

In this study, we first used HA-tyramine (HA-Tyr) as a carrier to synthesize ES/HA-Tyr and explore its sustained-release effects. Next, we studied the effects of ES/HA-Tyr on cell proliferation, invasion, and tube formation of HUVECs *in vitro*. Finally, we investigated the safety and efficacy of ES/HA-Tyr in Lewis lung cancer (LLC)-bearing mice, the possible benefits of combining it with RT, and the prospect of using it in the treatment of lung cancer.

## 2. Materials and methods

### 2.1. Reagents

ES (5 mg/mL, Mw 20 kDa) was provided by Shandong Simcere Medgeen Bio-Pharmaceutical Co. Ltd. (Yantai, China). HA (>95%, Mw = 90 kDa), tyramine hydrochloride (Tyr·HCl), N-hydroxysuccinimide (NHS), 1-ethyl-3-(3-dimethyl aminopropyl)-carbodiimide hydrochloride (EDCI), hydrogen peroxide (H_2_O_2_), horseradish peroxidase (HRP, 100 U/mg), and bovine testicular hyaluronidase were purchased from MeiLun Co. Ltd. (Dalian, China). Matrigel and 8-μm pore size cell culture inserts were purchased from Corning Incorporated Co. Ltd. (New York, USA). Human ES enzyme-linked immunosorbent assay (ELISA) kits were purchased from Andygene Co., Ltd. (Beijing, China). Platelet endothelial cell adhesion molecule-1 (CD31), type IV collagen, chondroitin sulfate proteoglycan 2 (NG2), vascular endothelial growth factor A (VEGF-A) polyclonal antibody, and hypoxia-inducible factor 1-alpha (HIF-1α) polyclonal antibody were purchased from Bioworld Technology Co. Ltd. (Nanjing, China). CD31, type IV collagen, NG2, VEGF-A, and HIF-1α were observed using an optical inverted Olympus IX73 microscope (Tokyo, Japan).

### 2.2. Cell culture and animals

LLC cells and HUVECs were cultured in DMEM (HyClone, Thermo Scientific, Waltham, MA) supplemented with 10% fetal bovine serum (FBS) (HyClone, Thermo Scientific, Waltham, MA) at 37 °C in a 5% CO_2_ incubator.

Seventy-two female C57 mice (4–6 weeks old) were purchased from the Chengdu Dashuo Experimental Animal Center (Chengdu, China) and maintained under specific pathogen-free conditions. All experiments were conducted in accordance with the guidelines of the Institutional Animal Care and Use Committee of the Southwest Medical University (Luzhou, China).

### 2.3. Synthesis of the ES/HA-Tyr conjugate

Briefly, HA (250 mg) and Tyr·HCl (202 mg) were dissolved in 50 mL of distilled water with continuous stirring at room temperature until the solution was clear. EDCI (479 mg) and NHS (290 mg) were added to activate the HA carboxyl groups. After an overnight reaction (pH = 4.5–5.0), the resulting HA-Tyr conjugate was dialyzed against 100 mM sodium chloride solution for 2 days, 25% ethanol solution for 1 day, and distilled water for 1 day. The purified conjugate solution was lyophilized for 3 days.

The dried HA-Tyr (15 mg) and ES (4 mg, which can be used in different concentrations) were dissolved in 1 mL phosphate-buffered saline (PBS) (pH = 7.3), followed by the addition of HRP and H_2_O_2_, 20 μL each. The final concentrations of HA-Tyr and ES were 15 mg/mL and 4 mg/mL, respectively. *In vitro,* the concentration of HA-Tyr was 15 mg/mL, and that of ES, 3 mg/mL.

### 2.4. In vitro release test of ES/HA-Tyr

*In vitro* ES release tests (ES [4000 pg] from ES/HA-Tyr hydrogels [1 mL]) were conducted in 10 mL PBS (pH = 7.3) at 37 °C using a shaking incubator. The prepared ES/HA-Tyr, ES/HA-Tyr + hyaluronidase-5, and ES/HA-Tyr + hyaluronidase-25 were placed in a test tube with 10 mL PBS. At selected time points, 200 μL of the solution was withdrawn and replaced with an equal volume of fresh buffer solution to maintain a constant total volume. ES concentration was measured using ELISA. Hyaluronidase-5 and hyaluronidase-25 are defined as PBS containing 5 unit/mL and 25 unit/mL of hyaluronidase, respectively.

### 2.5. Cell toxicity assay

An MTT assay was used to determine the anti-proliferative effects of HA-Tyr, ES/HA-Tyr, and ES on endothelial cells (Jiang et al., 2012; Ueda et al., 2016; Sun et al., 2018). Different concentrations of ES and ES/HA-Tyr were placed in a constant temperature water bath oscillator at 37 °C for 24 h. The extracts were collected and stored in a refrigerator at −20 °C for preservation. HUVECs were seeded into 96-well plates (5 × 10^3^ cells/well). After incubation overnight, endothelial cells were treated with extracts for 48 h, as described previously. The absorbance was measured at 490 nm.

### 2.6. Invasion assay

The effect of ES/HA-Tyr on HUVEC invasion was evaluated via the transwell assay (Jiang et al., 2012; Sun et al., 2018). Inserts of 8-μm pore size, coated with Matrigel, were placed in wells of a 24-well plate. HUVECs (2 × 10^4^ cells/insert) were plated to the upper chamber of the inserts without FBS. PBS, ES, HA-Tyr, or ES/HA-Tyr was added in the bottom chamber, supplemented with 10% FBS. After incubation at 37 °C for 48 h, the cells on the upper surface of the filter were removed with a cotton swab. The cells attached to the lower part of the filter were fixed, stained, and quantified using an optical inverted microscope (×200) (Olympus IX73 microscope, Tokyo, Japan).

### 2.7. Tube formation assay

The tube formation assay was used to evaluate the effect of ES/HA-Tyr on endothelial cell tubulogenesis (Li et al., 2011; Xiao et al., 2015; Sun et al., 2018), which simulates the angiogenesis process where endothelial cells migrate and aggregate to form a closed lumen. Briefly, Matrigel was pre-melted at 4 °C, coated on 24-well plates (300 μL/well), and then incubated at 37 °C for 30 min for polymerization. HUVECs were seeded in the Matrigel-coated plates (2 × 10^5^ cells/well). PBS, ES, HA-Tyr, or ES/HA-Tyr was added to each well. After an 8-h incubation, the enclosed tubes formed by HUVECs were counted and photographed with an inverted fluorescence microscope (×100).

### 2.8. Tumor models and treatment regimens

The mice were subcutaneously injected in the dorsal right thigh with 0.1 mL of LLC cells (1 × 10^6^ cells/mL). When the tumor tissue grew to 100–200 mm^3^, the mice were randomly divided into six groups (*n* = 12 each): control, HA-Tyr, ES, ES/HA-Tyr, RT, and ES/HA-Tyr + RT. The HA-Tyr and ES/HA-Tyr (ES: 4 mg/mL) solutions were injected intra-tumorally 7 times (on day 1, 3, 5, 7, 9, 11, and 13) from the first day of treatment. The control (PBS: 0.1 mL) and ES (2 mg/mL) solutions were injected intraperitoneally for 14 days from the first day of treatment. RT was subjected to 6 MeV electron irradiation with a radiation dose of 10 Gy on day 5 before injection (source to skin distance, 70 cm). Tumor volumes were calculated according to the formula *V = a × b^2^ ×* 0.5, where *a* was the longest diameter and *b* was the maximum transverse diameter. The tumor volumes were measured up to 17 days with a vernier caliper, and then the mice were sacrificed, and tumors from each group were surgically excised, rinsed with PBS, wiped, weighed, and photographed (Meng et al., 2013; Zheng et al., 2015; Zhu et al., 2015; Tang et al., 2019).

### 2.9. Histopathology

The toxicity of the therapeutic agent was evaluated according to the visceral toxicity (Yan et al., 2017). The heart, liver, lung, and kidney tissues of mice bearing subcutaneous xenograft tumors were harvested at the end of the experiment on day 17. Samples were excised, fixed with 10% neutral phosphate-buffered formalin, and embedded in paraffin. Continuous sections were obtained and stained with hematoxylin and eosin (H/E) for histomorphometric analyses.

### 2.10. Enzyme-linked immunosorbent assay (ELISA) analysis

Mice sera from the eyeball and tumor were collected, and ES levels were detected using an ELISA kit according to the manufacturer’s recommendation (Ding et al., 2017; Yan et al., 2017).

### 2.11. Immunofluorescence

Primary tumors were double-stained using a whole-mount staining protocol for CD31 and NG2 (Liu et al., 2018; Li et al., 2016). Tissues were fixed in 4% paraformaldehyde for 24 h, paraffin-embedded, sectioned, dewaxed in xylene, and rehydrated through graded alcohols. Antigen retrieval was performed in citric acid buffer (pH = 6.0). Sections were blocked in 2% normal goat serum for 1 h and stained with primary antibodies at 4 °C overnight. Endothelial cells and pericytes of tumor vessels were identified by staining with combinations of two antibodies. Endothelial cells were labeled with rat monoclonal anti-CD31 (1:500), and pericytes were labeled with rabbit anti-mouse NG2 antibody (1:400). Tumor tissues were double-stained with anti-CD31 and anti-NG2 antibodies. The sections were then washed and incubated with rhodamine-conjugated goat anti-rat IgG (H + L) (1:50) or goat anti-rat IgG-FITC (1:200) for 40 min at room temperature. The level of pericyte coverage was presented as a percent of the length along CD31^+^ vessels. Images were photographed under a fluorescence microscope (Olympus, Japan) and analyzed using ImageJ software (Public domain).

### 2.12. Micro ^18^FMISO PET/CT imaging

Hypoxia in tumors was evaluated by measuring^18^FMISO uptake (Tang et al., 2019) through micro PET/CT scans one day following treatment using Inveon micro PET/CT (Siemens, Munich, Germany). The mice were injected with 50–80 mCi ^18^FMISO into their tail veins. After 90 min of ^18^FMISO administration, the mice were gas anesthetized with Isoflurane and then placed in a central PET ring field; PET/CT images were obtained using the following parameters: 80 kV, 500 mA, slice thickness 1.5 mm, and 10 min per bed position. The image plane with the largest tumor appearance was selected for analysis, and the region of interest (ROI) was manually drawn across the entire tumor. Tracer uptake values of the tumors were measured in attenuation-corrected lateral chromatographic sections by calculating standard uptake values (SUVs) measured by ROI.

### 2.13. Immunohistochemistry (IHC)

The immunohistochemistry was used to determine the expression of HIF-1α and VEGF-A expression in xenograft tumors (Wu et al., 2014; Zhu et al., 2015; Ueda et al., 2016). Paraffin-embedded tumor tissue sections were deparaffinized in xylene, rehydrated in graded ethanol, and rinsed twice with PBS. Endogenous peroxidase activity was blocked by incubating sections with 3% H_2_O_2_ in the dark for 15 min. The sections were then incubated overnight at 4 °C with polyclonal antibodies to VEGF-A and HIF-1α (1:500). After washing with PBS, the slides were incubated with an anti-rabbit secondary antibody (diluted 1:100) for 1 h at room temperature. Finally, the slides were visualized by incubation with 3,3′-diaminobenzidine (DAB) and counterstained with hematoxylin (37%). Images were photographed under a light microscope (Olympus, Japan) and measured using ImageJ software.

### 2.14. Statistical analysis

The mean ± standard deviation (SD) from triplicate assays was calculated, and the differences between treatment groups were determined using a one-way analysis of variance (ANOVA) test with post-hoc contrasts by the least significance difference test. Statistical analysis was performed using SPSS statistics 17.0 software (SPSS Inc., Chicago, IL) and Prism 7.0 software (GraphPad, La Jolla, CA). *p* < .05 was considered statistically significant.

## 3. Results

### 3.1. ES/HA-Tyr preparation and in vitro release

**Figure 1.**
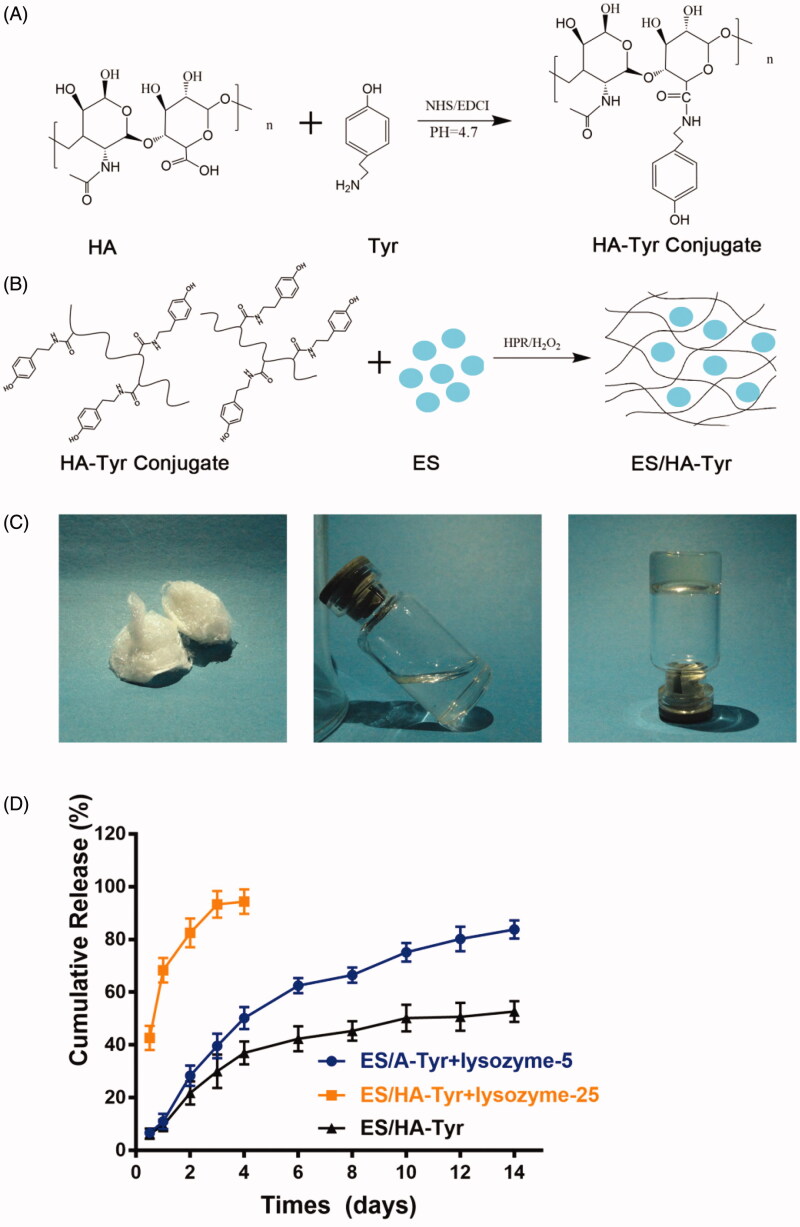
ES-HA-Tyr hydrogel formation process and release *in vitro*. (A) Synthesis of HA-Tyr conjugates. (B) Schematic representation for the synthesis of ES-HA-Tyr hydrogel. (C) Characterization of the injectable HA-Tyr hydrogel drug: the morphology of HA-Tyr after freeze-drying (C-left). A mixture of HA and ES before catalysis by HRP and H_2_O_2_ (C-middle). ES/HA-Tyr hydrogel formed after catalysis by HRP and H_2_O_2_ (C-right). (D) Cumulative release of ES from ES/HA-Tyr, ES/HA-Tyr + lysozyme-5, and ES/HA-Tyr + lysozyme-25. Data are expressed as means ± SD (*n* = 3). SD: standard deviation.

In this study, we successfully synthesized HA-Tyr conjugates by catalysis of EDCI and NHS (Figure 1(A)), and the freeze-dried HA-Tyr conjugates were white flocculent (Figure 1(C-left)). ES and HA-Tyr conjugates in deionized water were transparent, colorless, and fluid (Figure 1(C-middle)), and they formed ES/HA-Tyr under the catalysis of HRP and H_2_O_2_ (Figure 1(B)). ES/HA-Tyr had a colorless, transparent, and non-fluid semi-solid nature (Figure 1(C-right)).

*In vitro* release profiles of ES from the ES/HA-Tyr hydrogel drug were further analyzed, and the results are presented in Figure 1(D). For ES/HA-Tyr, the sudden release of ES was observed within the first 4 days, followed by an extremely slow-release, and the cumulative release of ES reached 52.7 ± 3.9% on the 14th day. For ES/HA-Tyr + lysozyme-5, sustained release of ES was observed, with a cumulative release of 83.83 ± 3.49% on the 14th day. For ES/HA-Tyr + lysozyme-25, we observed that the cumulative release of ES on day 4 reached 94.3 ± 4.63%. The release of ES from ES/HA-Tyr + lysozyme-25 was faster than from ES/HA-Tyr + lysozyme-5. Therefore, it was indicated that hyaluronidase contributed to the self-degradation of ES/HA-Tyr and released ES from the hydrogel.

### 3.2. Effects of ES/HA-Tyr on HUVECs in vitro

#### 3.2.1. ES/HA-Tyr was more cytotoxic to HUVECs

**Figure 2.**
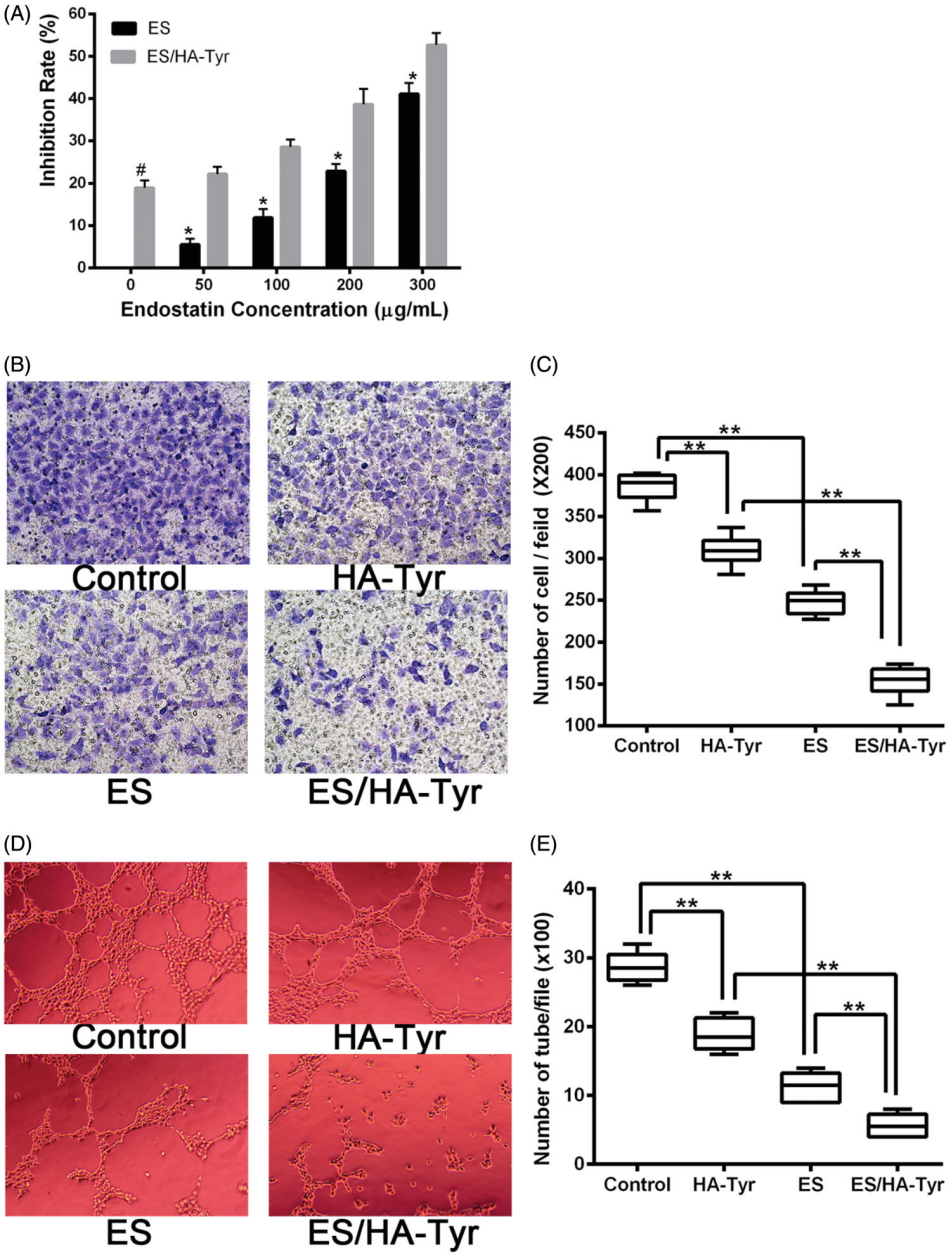
The inhibitory effect of ES/HA-Tyr on HUVECs *in vitro*. (A) Inhibition of ES and ES/HA-Tyr on endothelial cell proliferation *in vitro*. Data shown as means ± SD (*n* = 5). ES/HA-Tyr vs. ES *p < .05, ES/HA-Tyr vs. HA-Tyr ^#^*p* < .05. (B) Inhibition of the transwell invasion of endothelial cells after incubation with PBS, HA-Tyr, ES, and ES/HA-Tyr were photographed (×200). (C) Comparison of invaded cell numbers for PBS, HA-Tyr, ES, and ES/HA-Tyr in the transwell invasion assay. Data are expressed means ± SD (*n* = 10). **p < .01 between indicated groups. (D) The tube formations of HUVECs with PBS, HA-Tyr, ES, and ES/HA-Tyr photographed (×100). (E) Intact tubes enumerated and plotted. Data are expressed as means ± SD (*n* = 6), **p < .01 between indicated groups. HUVECs: human umbilical vascular endothelial cells; PBS: phosphate-buffered saline; SD: standard deviation.

The effect of ES and ES/HA-Tyr on the proliferation of HUVECs was assessed using the MTT assay. As shown in Figure 2(A), the inhabitation rate of ES/HA-Tyr at the concentrations of 50 μg/mL and 300 μg/mL were 22.2 ± 1.7%, and 52.7 ± 2.9%, respectively, while the rate for ES was 5.5 ± 1.4%, 41.1 ± 2.6%. As seen from the results, inhibition of HUVEC proliferation was stronger in ES/HA-Tyr than ES (*p* < .05; Figure 2(B)). The rate of inhibition was increased in a dose-dependent manner as the concentration increased.

#### 3.2.2. ES/HA-Tyr exhibited a more potent effect on HUVECs invasion

The transwell assay was used to determine the effect of ES, HA-Tyr, and ES/HA-Tyr on HUVEC invasion. The number of invaded HUVECs in the control, HA-Tyr, ES, and ES/HA-Tyr were 385.3 ± 16.1, 309.7 ± 16.3, 247.9 ± 13.2, and 153.5 ± 15.4, respectively. Compared with the control, the HUVEC invasion was significantly inhibited in ES and ES/HA-Tyr. Compared with ES, the invasion ratio in ES/HA-Tyr was significantly decreased (*p* < .01; Figure 2(B,C)). This demonstrated that ES/HA-Tyr was more potent at inhibiting HUVEC invasion.

#### 3.2.3. ES/HA-Tyr exhibited a stronger anti-angiogenic effect on HUVECs

The tube formation assay was used to evaluate the anti-angiogenic activity of ES, HA-Tyr, and ES/HA-Tyr on the neovascularization ability. As shown in Figure 2(D,E), the numbers of branches in the control, HA-Tyr, ES, and ES/HA-Tyr groups were 28.7 ± 2.2, 18.8 ± 2.3, 11.3 ± 2.1, and 5.7 ± 1.6, respectively. Compared with the control, ES and HA-Tyr significantly inhibited tube formation in HUVECs, and ES/HA-Tyr significantly enhanced the inhibitory effect compared with ES (*p* < .01). It demonstrated that ES/HA-Tyr induced more potent inhibition of tube formation in HUVEC cells.

### 3.3. ES/HA-Tyr decreased ES’s potential systemic toxic side effects in vivo

**Figure 3.**
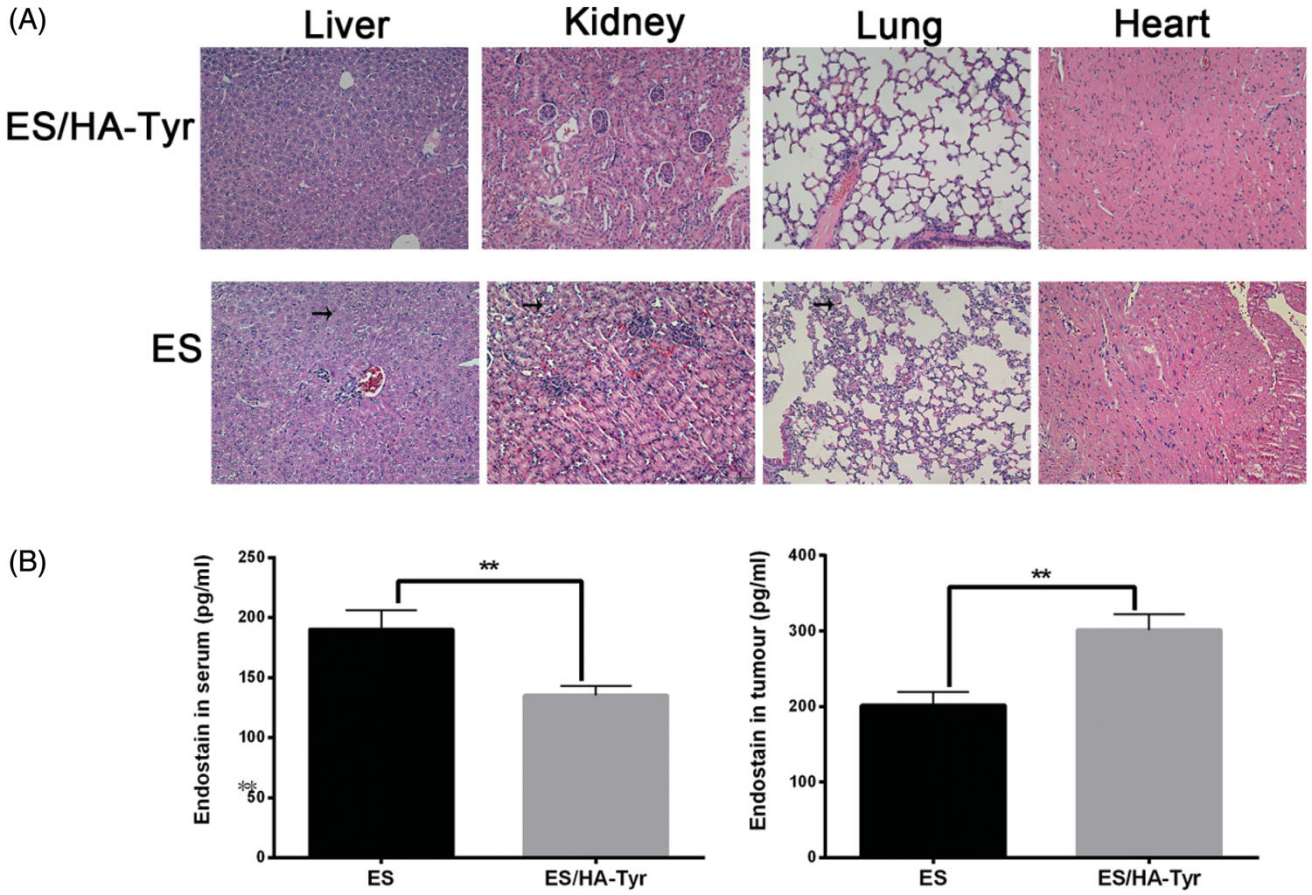
Systemic toxic side effects of ES/HA-Tyr evaluated in the tumor bearing mice. (A) Hematoxylin and eosin staining pictures of visceral tissue after ES and ES/HA-Tyr treatment, photographed (×200). (B) The concentration of ES in serum and tumor of mice in each group. Data are expressed as mean ± SD (*n* = 6). **p < .01. SD: standard deviation.

Visceral toxicity is the most common systemic toxic side effect of drugs. We assessed ES/HA-Tyr toxicity in tumor-bearing mice using H/E staining. As shown in Figure 3(A), compared with ES, ES/HA-Tyr showed dense and neatly arranged myocardial filaments, no damage of hepatocytes as the cytoplasm displayed no vacuolation, and normal alveolar and glomerular morphology, which suggests that ES/HA-Tyr decreased the potential systemic toxic side effects of ES.

The serum concentration of ES was assessed using an ELISA assay. As shown in Figure 3(B), in peripheral blood, the concentration of ES in ES/HA-Tyr (135.3 ± 3.2) was significantly lower compared with that of ES (190.4 ± 6.4) (*p* < .01), whereas in tumor tissues, the concentration of ES in ES/HA-Tyr (301.4 ± 8.4) was significantly higher (201.7 ± 7.1) (*p* < .01). ES/HA-Tyr could increase the local drug concentration of the tumor while reducing its concentration in the blood.

### 3.4. Effects of ES/HA-Tyr and ES/HA-Tyr + RT in vivo

#### 3.4.1. Anti-tumor effect of ES/HA-Tyr and ES/HA-Tyr + RT in LLC xenografts

**Figure 4.**
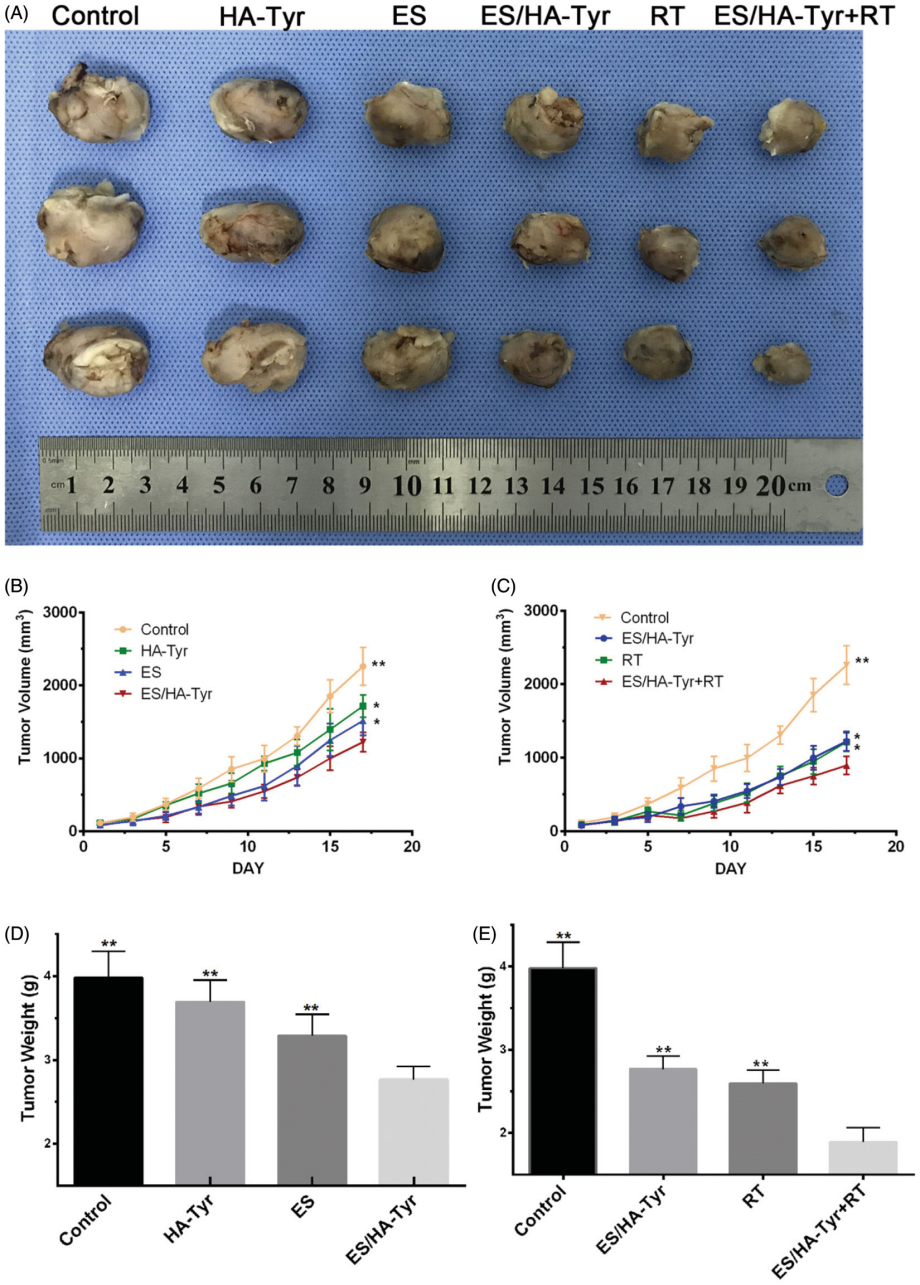
ES/HA-Tyr + radiotherapy (RT) inhibited tumor growth in the Lewis lung cancer model of lung cancer. (A) Tumor size of xenograft tumor. (B, C) Tumor volume–time graph. (D, E) Variations in tumor weight. Data are expressed as means ± SD (*n* = 6). *p < .05; **p < .01, ES/HA-Tyr compared with control, HA-Tyr, and ES, respectively. ES/HA-Tyr + RT compared with control, ES/HA-Tyr, and RT, respectively. SD: standard deviation.

Subcutaneous xenograft tumors in LLC mice were incubated in cages for 18 days. The tumor photograph, growth curve, and weight are shown in Figure 4. We found that ES/HA-Tyr led to a decrease in tumor volume and weight compared with that in ES (*p* < .01), while ES/HA-Tyr + RT led to a significant decrease in tumor volume and weight compared with that in ES/HA-Tyr and RT (*p* < .01). Taken together, ES/HA-Tyr + RT led to stronger tumor growth inhibition compared to that in either monotherapy.

#### 3.4.2. ES/HA-Tyr and ES/HA-Tyr + RT decreased angiogenesis and increased pericyte coverage in LLC

**Figure 5.**
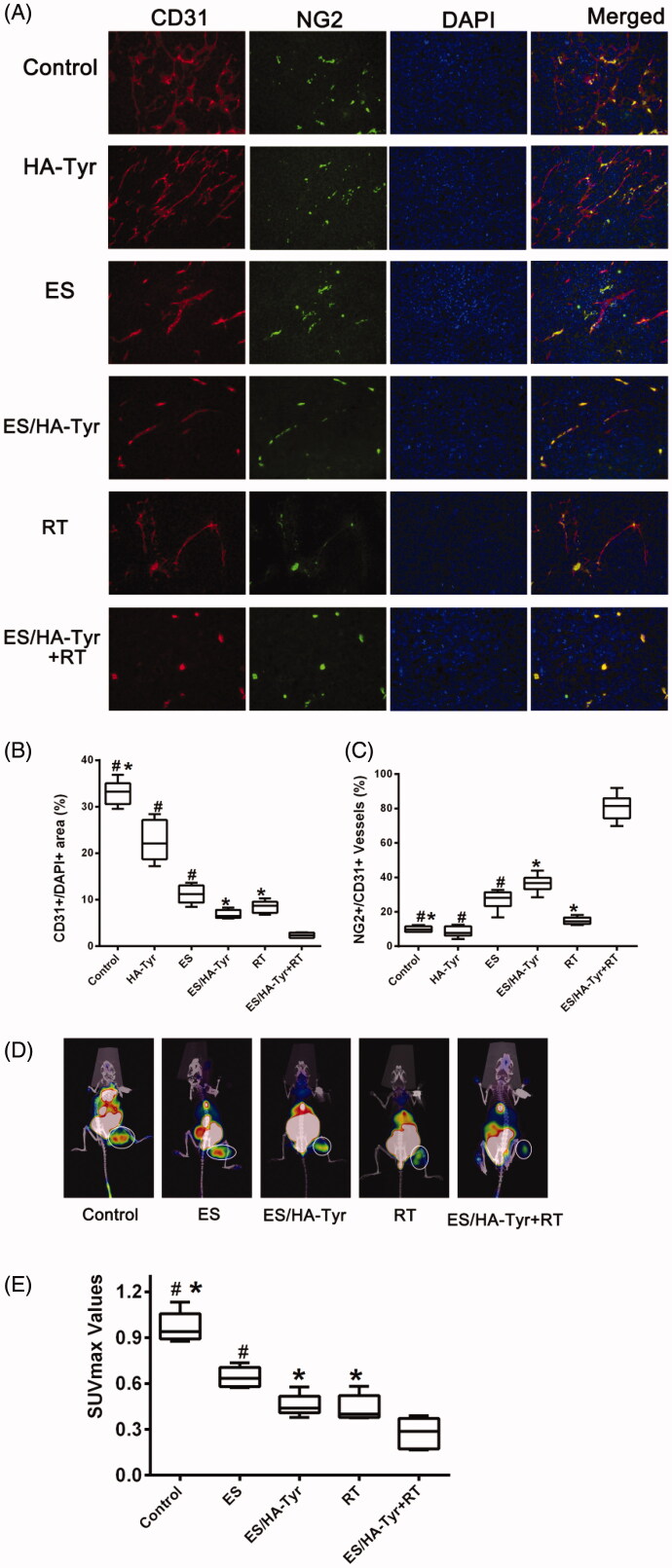
Tumor vasculature and hypoxia after treatment. (A) Fluorescence images of tumors showed CD31-positive endothelial cells (red), NG2-positive pericyte (green), DAPI-positive cell nucleus (blue), and merged images (yellow) photographed (×400). (B) Quantification of CD31-positive tumor vessels. (C) Quantification of the percentage of NG2 showed pericytes were increased during regression of endothelial cells in tumors. Data are expressed as means ± SD (*n* = 6). ES/HA-Tyr compared with control, HA-Tyr, and ES, respectively. ES/HA-Tyr + RT compared with control, ES/HA-Tyr, and RT, respectively. *p < .05, *p* < .05. (D) Representative images of the mice after the last treatment. (E) The SUVmax value of ROI in tumors. ES/HA-Tyr + RT compared to control, ES, ES/HA-Tyr, and RT, respectively. Data are expressed as means ± SD (*n* = 5). *p < .05, **p < .01. CD31, Platelet endothelial cell adhesion molecule-1; DAPI, 4′,6-diamidino-2-phenylindole; NG2, chondroitin sulfate proteoglycan 2. SD: standard deviation.

Immunofluorescent staining with CD31 was used to investigate whether the tumor vascularization and organization were modified after treatment. CD31 staining revealed that the vessels in the control group were chaotic and deformed, whereas tumor vessels that survived after 7 times of treatment with ES/HA-Tyr were less irregular, less tortuous, and had fewer branches and sprouts (Figure 5(A)). As shown in Figure 5(B), measurements of the vascularity of the tumors, we use CD31 as a marker of the endothelium. In Figure 5(C), we evaluated pericyte coverage in tumors by measuring NG2+/CD31+ vessels (%), with CD31 as a marker of the endothelium and NG2 as a marker of pericytes.

ES/HA-Tyr + RT (2.3 ± 0.5) revealed a conspicuous reduction in CD31 immunoreactivity compared with that in ES (11.2 ± 1.9) and ES/HA-Tyr (6.8 ± 0.9) (*p* < .05, Figure 5(B)). Meanwhile, ES/HA-Tyr + RT (80.7 ± 7.5) significantly increased in pericyte coverage compared with that in ES (27.1 ± 5.8) and ES/HA-Tyr (36.5 ± 5.0) (*p* < .05, Figure 5(C)). ES/HA-Tyr + RT significantly reduced angiogenesis while promoting vascular normalization.

#### 3.4.3. ES/HA-Tyr and ES/HA-Tyr + RT decreased LLC tissue hypoxia

Tumor cell hypoxia was assessed by Micro ^18^FMISO PET/CT uptake in LLC xenografts. Representative ^18^FMISO PET/CT images and the maximal standardized uptake value (SUVmax) of tumor-bearing mice are shown in Figure 5(D,E). As shown in Figure 5(E), the SUVmax value was reduced significantly in ES/HA-Tyr + RT (0.3 ± 0.1), followed by that in ES/HA-Tyr (0.5 ± 0.1), ES (0.6 ± 0.1), RT (0.44 ± 0.09), and control (1.0 ± 0.1) (*p* < .05). The lowest SUVmax value in the ES/HA-Tyr + RT group indicated a superior decrease in LLC tissue hypoxia with the combination therapy.

#### 3.4.4. The increased radio-response with ES/HA-Tyr and ES/HA-Tyr + RT are associated with decreased expression of HIF-1α and VEGF-A

**Figure 6.**
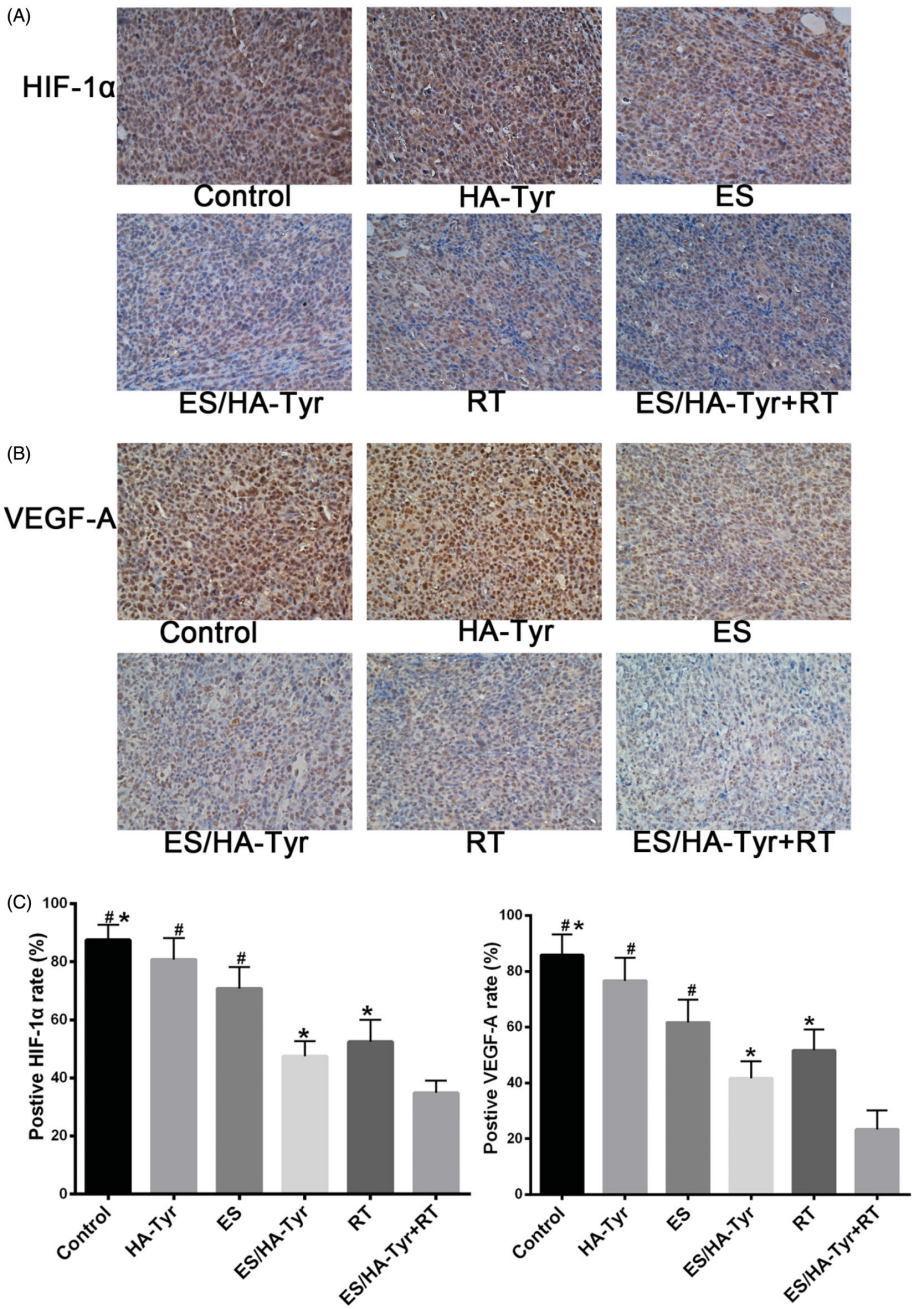
HIF-1α and VEGF-A expression in xenograft tumors from different groups. (A) Representative immunohistochemistry (IHC) images showing HIF-1α expression in tumor tissues original magnification, photographed (×400). (B) Representative IHC images showing VEGF-A expression in tumor tissues. Original magnification, photographed (×400). (C) Histogram showing the percentage of HIF-1α and VEGF-A positive cells in each group. ES/HA-Tyr compared with control, HA-Tyr, and ES, respectively. ES/HA-Tyr + RT compared with control, ES/HA-Tyr, and RT, respectively. *p < .05; ^#^*p* < .05. HIF-1α: hypoxia-inducible factor 1-alpha; VEGF-A: vascular endothelial growth factor A; SD: standard deviation.

The effect of ES/HA-Tyr + RT on tumor hypoxia and angiogenesis was assessed by the expression of HIF-1α (Figure 6(A)) and VEGF-A (Figure 6(B)) in xenografted tumor tissues. The proportion of HIF-1α positive cells (Figure 6(C-left)) was significantly lower in ES/HA-Tyr + RT (34.8 ± 4.4) compared with that in ES/HA-Tyr (47.5 ± 5.2) and ES (70.8 ± 7.4) (*p* < .05). The proportion of VEGF-A positive cells (Figure 6(C-right)) was significantly lower in the ES/HA-Tyr + RT group (27.5 ± 5.2) compared with that in the ES/HA-Tyr (42.5 ± 5.2) and ES groups (61.7 ± 8.2) (*p* < .05). Tumors in the ES/HA-Tyr + RT group showed significantly lower expression levels of HIF-1α and VEGF-A compared with those in either monotherapy group. This suggests that ES/HA-Tyr + RT has a better radio-response.

## 4. Discussion

In China, the morbidity of tumor is increasing, and RT and anti-angiogenic therapy are commonly used. However, existing anti-angiogenic drugs are often administered using systemic administration, which increases tumor drug concentration and amplifies systemic toxicity. Therefore, we synthesized anti-angiogenic hydrogel drugs, hoping to inject drugs locally into the tumor through the needle channel after chest biopsy. We studied whether this could not only increase the anti-tumor effect but also reduce the systemic toxicity compared with systematic administration.

It is crucial for an injectable hydrogel system to crosslink rapidly because slow gelation causes gel precursors away from the injection site and leakage of the drugs encapsulated in the gel (Gupta et al., 2006; Lee et al., 2008). HA is a natural hydrogel with slow gelation so we choose tyrosine as the functional molecule to accelerate the gelation of the hydrogel. HA-Tyr was formed by oxidative coupling reaction of tyrosine (Tyr); which sustained-release drugs confirmed (Kurisawa et al., 2005; Lee et al., 2009; Xu et al., 2015). This was a drug delivery system developed specifically for protein and peptide drugs, and its biosafety was also validated (West & Kumar, 1989). In this study, we have successfully synthesized ES/HA-Tyr in our study (Figure 1(A–C)) through some modifications in the preparation of HA-Tyr. These results revealed that ES/HA-Tyr can release ES continuously *in vitro*, and the release and degradation rates of ES/HA-Tyr depended on hyaluronidase concentration (Figure 1(D)). The incomplete release of ES in ES/HA-Tyr may be due to the electrostatic interactions between ES and the carboxyl groups of HA-Tyr. Studies have indicated that protein drugs easily interacted with hydrogel groups (Kurisawa et al., 2005; Lee et al., 2009).

Due to its endothelium-specificity, ES can inhibit HUVEC proliferation, invasion, migration, and tube formation, and induce apoptosis *in vitro* (Li et al., 2011; Xiao et al., 2015; Sun et al., 2018). We confirmed that ES/HA-Tyr was superior to ES in cytotoxicity, invasion, and tube formation inhibition of HUVECs *in vitro* at the same concentration (Figure 2). Thus, HA-Tyr had a certain inhibitory effect on HUVECs. Additionally, the high molecular weight of HA can inhibit endothelial cells when the concentration is >100 μg/mL (Goel et al., 2011), thereby inhibiting tumor growth. However, the exact molecular mechanisms exerted by ES/HA-Tyr and HA-Tyr in their anti-angiogenic efficacy remain unclear and should be investigated further.

Solid tumors are known for their low uptake of anti-angiogenic drugs. If we want to increase the concentration of drugs in tumor tissue, there are different degrees of potential systemic toxicity side effects, which can lead to organ damage. Thus, we evaluated the systemic toxicity of ES/HA-Tyr *in vivo* using ELISA and histopathology assessments. The results showed that ES/HA-Tyr can increase local drug concentration while reducing blood concentration and causing lower systemic toxicity (Figure 3).

An abnormal tumor vascular system has high permeability and tortuosity, which damages the blood supply of tumor tissues and leads to hypoxia and acidity of the tumor microenvironment. Changes in the tumor microenvironment promote tumor invasion and metastasis, whereas hypoperfusion limits the entry of therapeutic drugs into the tumor, resulting in insensitivity to chemotherapy/RT^4^. Pericytes are considered as mediators of angiogenesis and metastasis in tumors (Carmeliet & Jain, 2000; Meng et al., 2013; Shi et al., 2019). They typically encapsulate endothelial cells, coordinate intercellular signals, establish direct cellular contact, and support endothelial cell integrity, stability, and maturation. We used LLC to establish a subcutaneous tumor model in C57 mice and used an intra-tumor injection of ES/HA-Tyr to prove that ES/HA-Tyr has better anti-tumor effects than ES (Figure 4(A,B,D)). Fluorescence staining of tumor tissues by CD31/NG2 (Figure 5(A–C), *p* < .05) and immunohistochemical staining of tissues by VEGF-A (Figure 6(B,C-right), *p* < .05) revealed that ES/HA-Tyr can more effectively inhibit tumor blood vessel growth and promote tumor vessel normalization than ES. Through the examination of tumor hypoxia by 18FMISO (Figure 5(D,E), *p* < .05) and HIF-1α (Figure 6(A,C-left), *p* < .05), we found that ES/HA-Tyr can more effectively reduce hypoxia in tumor tissues than ES.

As RT is a common method of anti-tumor therapy, the radiosensitization effect of ES has been reported (Jiang et al., 2012; Peng et al., 2012; Meng et al., 2013). Therefore, we explored the effect of ES/HA-Tyr combined with RT in LLC. We chose high dose of RT because we had achieved certain results in the field of high dose RT, which will be widely used in clinical practice (42). Fluorescence staining of tumor tissues by CD31/NG2 (Figure 5(A–C), *p* < .05) and immunohistochemical staining of tissues by VEGF-A (Figure 6(B,C-right), *p* < .05) revealed that ES/HA-Tyr + RT can more effectively inhibit tumor blood vessel growth and promote tumor vessel normalization than either monotherapy. Through the examination of tumor hypoxia by ^18^FMISO (Figure 5(D,E), *p* < .05) and HIF-1α (Figure 6(A,C-left), *p* < .05), we also found that ES/HA-Tyr + RT can more effectively reduce hypoxia than either monotherapy in tumor tissues. These results suggest that ES/HA-Tyr + RT exhibited a better anti-tumor effect.

In this study, we synthesized ES/HA-Tyr, and its sustained-release and anti-tumor characteristics *in vivo* and *in vitro* were verified. Furthermore, we established that ES/HA-Tyr combined with RT has a better anti-tumor effect. However, this study has some limitations. First, we only simulated the release of drugs in the tumor environment; we do not know the impact of the real tumor microenvironment on drug release. Therefore, further release experiments *in vivo* are needed. Second, the experimental effect has only been verified on one animal model and needs to be further studied for other tumor types. The combination of other treatment methods also needs to be explored in future studies. Third, intratumor administration can only be used in the treatment of solid tumors with a certain volume and is an invasive treatment method. However, we injected ES/HA-Tyr immediately after the CT-guided needle biopsy, which can reduce secondary injury to the patient, and the sustained drug release can greatly extend the patient’s administration cycle. We believe that ES/HA-Tyr has clinical applications in anti-tumor therapy.

## 5. Conclusions

Briefly, this study revealed that ES/HA-Tyr had a significant, sustained release and can continuously inhibit the proliferation, invasion, and tube formation of endothelial cells. Furthermore, intra-tumor injection of ES/HA-Tyr effectively increased the local ES drug concentration and reduced the serum concentration, which further reduced the systemic side effects of ES. Last, our data revealed that ES/HA-Tyr and ES/HA-Tyr + RT reduced abnormal tumor vascular growth, improved pericyte coverage, and reduced hypoxia, thus increasing the efficacy of RT. ES/HA-Tyr + RT also showed a better anti-tumor effect in the LLC xenotransplantation model.
